# Prognostic Value of the Albumin-Bilirubin Grade for the Prediction of Post-Hepatectomy Liver Failure: A Systematic Review and Meta-Analysis

**DOI:** 10.3390/jcm10092011

**Published:** 2021-05-08

**Authors:** Giovanni Marasco, Luigina Vanessa Alemanni, Antonio Colecchia, Davide Festi, Franco Bazzoli, Giuseppe Mazzella, Marco Montagnani, Francesco Azzaroli

**Affiliations:** 1 IRCCS Azienda Ospedaliero-Universitaria di Bologna, 40138 Bologna, Italy; vanessaalemanni1@gmail.com (L.V.A.); franco.bazzoli@unibo.it (F.B.); marco.montagnani@unibo.it (M.M.); francesco.azzaroli@unibo.it (F.A.); 2 Department of Medical and Surgical Science, University of Bologna, 40126 Bologna, Italy; davide.festi@unibo.it (D.F.); giuseppe.mazzella@unibo.it (G.M.); 3 Gastroenterology Unit, University Hospital Borgo Trento, 37100 Verona, Italy; antonio.colecchia@aovr.veneto.it

**Keywords:** hepatocellular carcinoma, liver surgery, hepatectomy, liver failure, albumin-bilirubin, ALBI, PHLF

## Abstract

(1) Introduction: Liver resection (LR) for hepatocellular carcinoma (HCC) is often burdened by life-threatening complications, such as post-hepatectomy liver failure (PHLF). The albumin-bilirubin (ALBI) score can accurately evaluate liver function and the long-term prognosis of HCC patients, including PHLF. We aimed to evaluate the diagnostic value of the ALBI grade in predicting PHLF in HCC patients undergoing LR. (2) Methods: MEDLINE, Embase, and Scopus were searched through January 17th, 2021. Studies reporting the ALBI grade and PHLF occurrence in HCC patients undergoing LR were included. The Odds Ratio (OR) prevalence with 95% confidence intervals (CI) was pooled, and the heterogeneity was expressed as I^2^. The quality of the studies was assessed using QUADAS-2 (Quality Assessment of Diagnostic Accuracy Studies). (3) Results: Seven studies met the inclusion criteria and were included in the analysis. A total of 5377 patients who underwent LR for HCC were considered, of whom 718 (13.4%) developed PHLF. Patients with ALBI grades 2 and 3 before LR showed increased rates of PHLF compared to ALBI grade 1 patients. The pooled OR was 2.572 (95% CI, 1.825 to 3.626, *p* < 0.001), with substantial heterogeneity between the studies (I^2^ = 69.6%) and no publication bias (Begg’s *p* = 0.764 and Egger’s *p* = 0.851 tests). All studies were at a ‘low risk’ or ‘unclear risk’ of bias. Univariate meta-regression analysis showed that heterogeneity was not dependent on the country of study, the age and sex of the participants, the definition of PHLF used, the rate of patients in Child–Pugh class A or undergoing major hepatectomy. (4) Conclusions: In this meta-analysis of published studies, individuals with ALBI grades of 2 and 3 showed increased rates of PHLF compared to ALBI grade 1 patients.

## 1. Introduction

Hepatocellular carcinoma (HCC) represents the second cause of cancer-related death worldwide [1]; in 90% of cases, it develops with underlying liver disease, leading to a relevant burden in morbidity and mortality in patients affected by chronic liver disease [2]. Despite several techniques for HCC management that have been developed in the last decades, liver resection (LR) still represents the main curative treatment offering the best outcome [3]. LR is often burdened by life-threatening complications, such as post-hepatectomy liver failure (PHLF) [4]. 

PHLF has been described with an incidence ranging from 8% to 12% [5]; this variability is related to different PHLF definitions and the severity of the underlying liver disease, the extent of surgery, and the intraoperative course [4]. The current International Study Group of Liver Surgery (ISGLS) definition for PHLF represents the current standard method for its diagnosis [5,6], defined as an acquired deterioration of the liver functions characterized by an increased International Normalized Ratio (INR) and hyperbilirubinemia after postoperative day 5 [7]. 

Thus, an accurate pre-operative assessment of patients undergoing LR is required to overcome the PHLF risk, through the evaluation of liver function and the assessment of portal hypertension [8,9,10]. Several markers have been previously proposed for this purpose, such as the Child–Pugh, liver stiffness measurement, and volumetric imaging for future liver remnant assessment, which are typically performed in the pre-operative work [9,11]. In Eastern countries a large use of the indocyanine green clearance (ICG) has been reported [12]. 

However, these evaluations are often expensive, time consuming, or inaccurate in providing a precise estimation of post-operative hepatic functional recovery [13]. Recently, the albumin-bilirubin (ALBI) score, a new non-invasive tool to evaluate liver function and predict survival in HCC patients [14], showed promising results in predicting the long-term prognosis of chronic liver disease [15] and HCC patients [16], including PHLF prediction. However, no definitive results are available for this latter outcome and, consequently, the ALBI grade has not been completely endorsed in the pre-operative assessment of HCC patients undergoing LR. Thus, we performed a systematic review with meta-analysis to evaluate the diagnostic value of the ALBI grade in predicting PHLF in HCC patients undergoing LR.

## 2. Materials and Methods

We performed a systematic review and meta-analysis following the recommendations of the Cochrane Collaboration Diagnostic Test Group [17] and according to the PRISMA (Supplementary Material 1) (Preferred Reporting Items for Systematic Reviews and Meta-Analyses) guidelines [18].

### 2.1. Search Strategy and Study Selection

We searched on MEDLINE via PubMed, Ovid Embase, and Scopus, to identify relevant articles published up to 17 January 2021. The electronic search of the literature was conducted using the following keywords: ‘ALBI’ or ‘albumin bilirubin’, ‘PHLF’ or ‘post-operative liver failure’ or ‘post hepatectomy liver failure’, and ‘liver resection’ or ‘hepatectomy’ or ‘hepatic resection’. 

The search was extended until 2015, when the first article published on ALBI scores was published [14]. In addition, the abstracts of the conference proceedings of Digestive Diseases Week, United European Gastroenterology Week, International Liver Congress, American Association for the Study of Liver Diseases Meeting, and Asian Pacific Association for the Study of the Liver Congress for the same period were searched electronically and by hand. 

The complete search strategies are reported in Supplementary Material 2. There were no limitations on the type of study, publication date, or manuscript language. Two reviewers (LVA and GM) independently performed the initial screening and selection, based on titles and abstracts. Eligible full-text articles were separately evaluated by the two authors; in the case of discrepancies, they were resolved through discussion with a third reviewer (FA).

Studies were selected and included in final analysis when they met the following criteria: studies conducted on patients affected by chronic liver diseases undergoing LR for HCC, reporting data on the ALBI grade in patients developing PHLF or not. All etiologies for liver disease were included. In the presence of studies reporting cohorts who underwent LR for HCC and other malignancies, further data on only HCC patients were requested from the authors; in the case of no response, we established a minimum of 90% of HCC within a study population to include the paper in our analysis. 

Only studies reporting PHLF diagnosed according to ISGLS criteria [7] were included, as this classification has been widely endorsed [5]. As recommended by ISGLS [7], PHLF had to be diagnosed in the case of increased serum International Normalized Ratio (INR) and concomitant hyperbilirubinemia, after 5 postoperative days. The severity of PHLF was therefore graded as: grade A PHLF, requiring no specific treatment; grade B PHLF requiring essential non-invasive treatment (transfusion support, albumin supplementation, and diuretic therapy); grade C PHLF requiring invasive procedures, including mechanical ventilation, hemodialysis, or extracorporeal liver support [7]. 

PHLF grades B and C are, thus, considered clinically significant [7]. The ALBI score is based on the serum albumin and total bilirubin levels, calculated with the formula: (log10 bilirubin [µmol/L] × 0.66) + (albumin [g/L] × −0.0852). This score is further categorized into three different grades for rapid clinical use: ALBI 1 (≤−2.60), ALBI 2 (>−2.60 to ≤−1.39), and ALBI 3 (>−1.39) [14]. 

We included only studies reporting the number of PHLF cases for each ALBI grade. For studies reporting the ALBI score instead of the grade, we contacted the authors in order to collect the missing data. Studies were excluded if they did not meet the inclusion criteria or when essential information was missing in the available manuscript or could not be obtained from the authors. 

### 2.2. Data Extraction and Quality Assessment

Two authors (LVA and GM) independently extracted relevant data on the publication, study methods, and results using a standardized data extraction form. The following items were extracted from each study: type of study, year of publication, country, study design, total number of patients enrolled, age and sex of the participants, Child–Pugh classification, main etiology of liver disease, the extent of LR, the number of PHLF cases classified by the severity degrees (PHLF A, B, and C), and ALBI grade groups. 

If multiple publications on a same cohort were found, the latest and most complete publication was considered. Subsequently, the methodological quality of the included studies was separately assessed by two reviewers (LVA and GM), according to the Quality Assessment of Diagnostic Accuracy Studies (QUADAS-2) tool [19] (Supplementary Material 3). QUADAS-2 is an evidence-based tool consisting of 14 items phrased as questions, each of which are scored a “yes”, “no”, or “unclear”, examining the presence of bias in the study. Disagreements were resolved through discussion or arbitration by a third reviewer (FA), when necessary.

### 2.3. Statistical Analysis

The rates of PHLF in patients with ALBI grade 1 and ALBI grades 2 and 3 were extracted from all studies. As we expected that only a small amount or no patients undergoing LR would have severely impaired liver function according to ALBI grade 3, we decided to consider for statistical analyses ALBI grades 2 and 3 compared to ALBI grade 1. The pooled Odds Ratios (ORs) with corresponding 95% Confidence Intervals (CI) and *p* were calculated to assess the association between the ALBI grade and PHLF occurrence in patients with HCC. 

Heterogeneity across the studies was assessed using the Higgins I^2^ statistics. The value of I^2^ describes the percentage of variability in point estimates due to heterogeneity rather than to sampling error: low-moderate for I^2^ < 50% and high for I^2^ ≥ 50% [20]. If there was no heterogeneity (<50%, *p* > 0.1), the fixed-effect model was used; otherwise, the random-effect model was applied. The ALBI grades 2 and 3 were closely associated with PHLF when the OR > 1. The publication bias was measured by Begg’s test and Egger’s test with a graph; a *p* value < 0.05 indicated a significant small size study effect. 

Briefly, Begg’s test and Egger’s test are based on the statistical evaluation of a funnel plot, which shows the effect sizes plotted against their standard errors, instead of the visual evaluation of asymmetry. While Begg’s test examines the correlation between the effect sizes and their variances, Egger’s test regresses the standardized effect sizes on their precisions. The Duval and Tweedie [21] non-parametric ‘trim and fill’ method was also used, accounting for publication bias in the meta-analysis [22]. Pooled ORs following adjustment for the publication bias using the ‘trim and fill’ method are reported. Subgroup analyses were conducted after excluding studies with possible sources of heterogeneity (studies including subgroups of patients other than HCC or including only PHLF B and C as the case groups). 

As part of the sensitivity analysis, the impact of confounding covariates (country, age of participants, sex, rate of patients in Child–Pugh class A or undergoing major hepatectomy, definition of PHLF (PHLF vs. clinically significant PHLF)) on the meta-analytic results was evaluated using meta-regression analysis [23], reporting β coefficient ± standard error (SE). Since a low number of studies was found, the *p* values were also recalculated using Monte Carlo permutation [24] with 5000 permutations to obtain sufficient precision [25]. All analyses were carried out using STATA statistical software (Stata Corp., College Station, TX, USA). 

## 3. Results

### 3.1. Study Selection

The electronic and manual searches provided 215 records; after duplicate elimination, 180 studies went through screening based on the title and abstracts. After the first screening, 25 records (19 [26,27,28,29,30,31,32,33,34,35,36,37,38,39,40,41,42,43,44] full-text papers and six [45,46,47,48,49,50] abstracts) remained for fully eligibility evaluation, of which one [29] was found during the manual search. 

**Figure 1:**
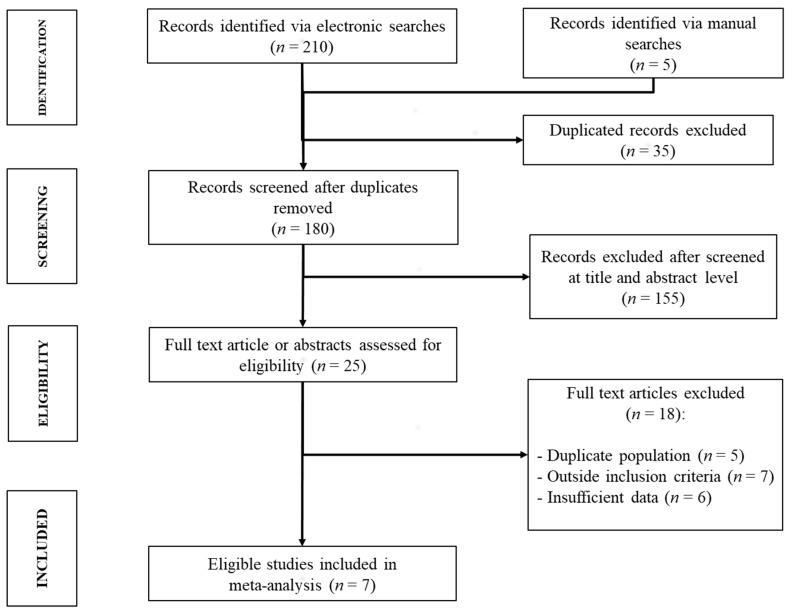
The Preferred Reporting Items for Systematic Reviews and Meta-Analyses (PRISMA in Supplementary Material 1) flow diagram of the systematic literature search and the studies included in the meta-analysis.

Among them, six [26,27,30,34,47,48] were excluded from the meta-analysis due to insufficient data or no response by the study authors; five [28,35,46,49,50] studies were excluded since the reporting cohorts were already included or best characterized in more recent studies; one study [42] was not pertinent since it was performed on patients undergoing extrahepatic surgery; a further six studies [31,32,33,37,39,41] included large groups of patients undergoing LR for malignancies other than HCC and/or did not use the ISGLS criteria [7] for PHLF diagnosis. Finally, a total of seven studies [29,36,38,40,43,44,45], all full text except one [45], met the eligibility criteria and were included in the meta-analysis, as shown in Figure 1.

### 3.2. Study Characteristics

The seven studies included [29,36,38,40,43,44,45] reported a total number of 5377 patients undergoing LR for HCC, of whom 718 (13.4%) developed PHLF, among which 502 (69.9%) were clinically significant. Only one study [44] included a negligible sub-group (9.2%) of patients who underwent LR for reasons other than HCC. Notably, two studies [38,44] reported only on PHLF grades B and C, while others [29,36,40,43,45] considered all grades of severity. The characteristics of the studies included are shown in Table 1.

In particular, five studies [36,38,40,43,45] were conducted in China, whereas two [29,44] were conducted in Italy. Five studies [29,36,40,44,45] reported the median age of the participants, which ranged from 52 [36,40] to 70 [29] years. In all studies, a greater rate of male patients were included, with the proportion ranging from 73.5% [44] to 88.8% [38]. All the studies had a retrospective design, with the exception of one prospective study [44]. Six studies [29,36,38,43,44,45] reported the extent of the hepatectomy. Notably, the rate of major hepatectomy ranged from 6.7% [44] to 38.6% [45]. 

Regarding liver function, the Child–Pugh A was reported in variables rates, ranging from 90.3% [36] to 100% [38], whereas the Child B was reported in rates ranging from 0% [38] to 9.7% [36]. Asian studies [36,38,40,43,45] presented mainly HBV-related liver disease (from 80.3% [45] to 88.9% [38]), while, in European studies, different etiologies were reported (from 40% [29] to 59% [44] HCV-related). Within the included studies, patients were categorized according to an ALBI grade as follows: grade 1 (3470 pts, 64.53%), grade 2 (1887 pts, 35.1%), and grade 3 (20 pts, 0.37%). The PHLF rates for each ALBI category were as follows: ALBI 1 (330 pts, 9.5%), ALBI 2 (376 pts, 19.9%), and ALBI 3 (12 pts, 60%).

### 3.3. Quality Assessment

The evaluation of the methodological quality of the included studies is reported in Supplementary Material 4 and in Table 2. The studies considered in the meta-analysis had an overall low risk of bias according to QUADAS-2. However, all studies presented ‘unclear risk’ concerning the risk of bias regarding the ‘reference test’ and the ‘flow and timing’. Indeed, in all studies, it was not reported whether the diagnosis of PHLF was blinded to the ALBI grade. The exact timing of the pre-operative functional tests was also not specified in the studies included. An ‘unclear risk’ of bias in the ‘patient selection’ was present in three studies [36,38,40]. Concerns regarding the applicability of the reference standard were raised for two studies [38,44], as they reported only clinically significant PHLF.

### 3.4. ALBI and PHLF Occurrence

**Figure 2:**
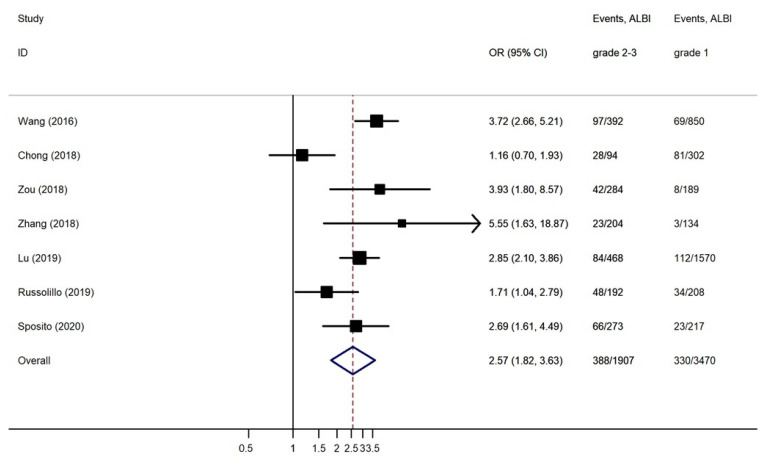
Forest plot of the pooled Odds Ratio (OR) for post-hepatectomy liver failure (PHLF) in ALBI grades 2 and 3 compared to ALBI grade 1. Events: PHLF/No PHLF; and CI: Confidence Interval.

**Figure 3:**
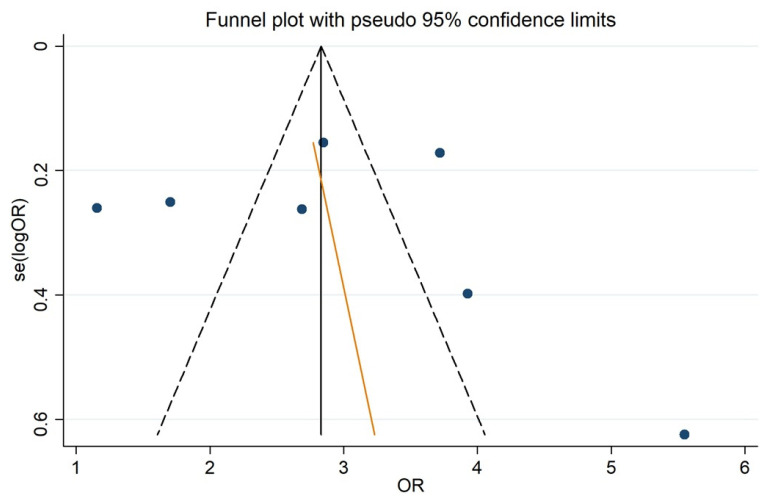
Funnel plot visual to asymmetry. Legend: OR: Odd Ratio. se(logOR): Standard Error of log OR. Dotted black line: the line of pseudo 95% confidence limits. Solid black line: the line of overall effect. Blue point: each study included. Orange line: Egger’s test.

Patients with ALBI grades 2 and 3 before LR showed increased rates of PHLF compared to ALBI grade 1 patients. The pooled OR using a was 2.572 (95% CI, 1.825 to 3.626) (Figure 2). This difference was statistically significant (*p* < 0.001). There was substantial heterogeneity between the studies (I^2^ = 69.6%). No publication bias was found using Begg’s (*p* = 0.764) and Egger’s (*p* = 0.851) tests (Figure 3). Due to an asymmetrical appearance of the funnel plot, the ‘trim and fill’ method was applied, indicating no missing studies. 

**Figure 4:**
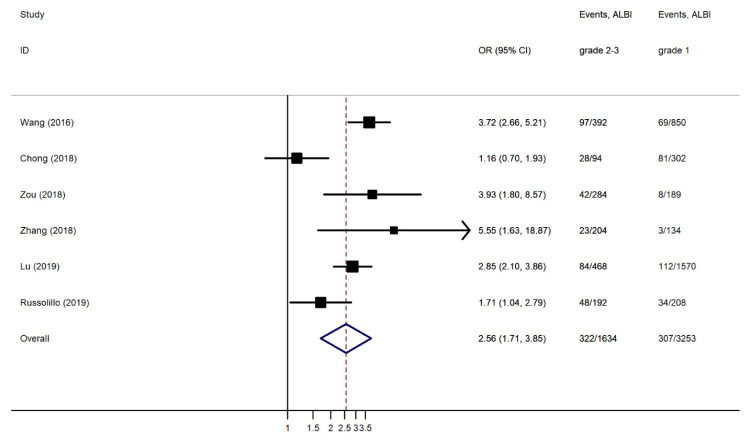
Forest plot of the pooled Odds Ratio (OR) for post-hepatectomy liver failure (PHLF) in ALBI grades 2 and 3 compared to ALBI grade 1 after removing one study that included a small subgroup of patients other than HCC. Events: PHLF/No PHLF; and CI: Confidence Interval.

**Figure 5:**
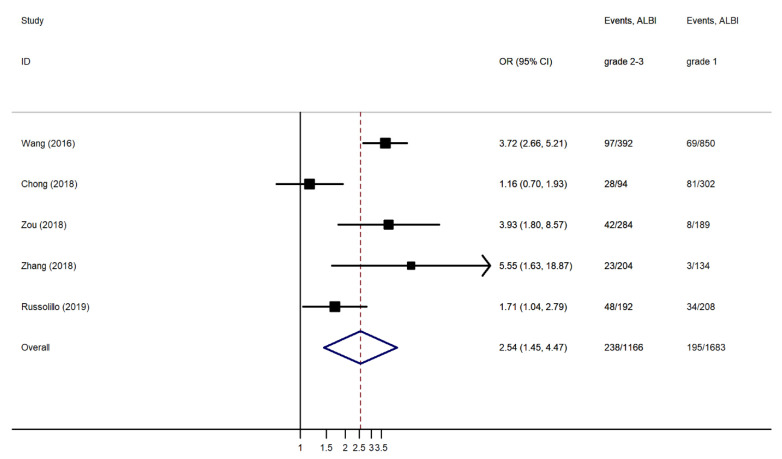
Forest plot of the pooled Odds Ratio (OR) for post-hepatectomy liver failure (PHLF) in ALBI grades 2 and 3 compared to ALBI grade 1 after removing two studies that included only PHLF B and C as case groups. Events: PHLF/No PHLF; and CI: Confidence Interval.

The re-estimated OR slightly increased but remained significantly different among the two groups (OR 2.997, 95% CI 2.193 to 3.801, *p* < 0.001). The subgroup analyses after removing one study [44], including a small subgroup of patients other than HCC (<10%) (OR 2.564, 95% CI 1.708 to 3.849, I^2^ 74.6%, *p* < 0.001) (Figure 4), and after removing two studies [38,44], including only PHLF B and C as the case groups (OR 2.543, 95% CI 1.446 to 4.471, I^2^ 79.3%, *p* < 0.001) (Figure 5), showed a slight variation in the ORs.

Univariate meta-regression analysis was used to explore and explain potential sources of heterogeneity among the studies. None of the variables assessed was able to explain the high heterogeneity found (Table 3) even after 5000 permutations.

## 4. Discussion

PHLF represents a major event in patients undergoing LR and mostly affects patients with chronic liver disease complicated by HCC development [4]. To date, the selection of patients undergoing LR according to the risk of post-operative complications, such as PHLF, is unsatisfactory [13]. This systematic review and meta-analysis included six studies reporting data on the ALBI grade in patients developing PHLF. The pooled data available from these studies showed that patients with ALBI grades of 2 and 3 had an increased rate of developing PHLF compared to ALBI grade 1 patients (OR of 2.572). 

To our knowledge, this is the first meta-analysis aiming to assessing the association between the ALBI score and PHLF occurrence using the QUADAS-2 tool for a correct evaluation of the methodological quality of the studies included. The association between PHLF and ALBI is undoubtedly explained by the accuracy of the ALBI grade in non-invasively mirroring the liver function [14] even in patients with mild or early stage liver disease. The prevention of PHLF is achievable mostly by a careful liver function assessment in preoperative examinations [3]. 

The Child–Pugh classification remains the most applied method for the evaluation of the liver reserve in the preliminary evaluations for LR [3]. However, in recent years, concerns regarding the adequacy of the Child–Pugh classification have emerged due to the subjectivity and insufficient ability in stratifying the individual risks of patients with mild severity liver diseases [14,51,52]. Instead, the ALBI grade showed a greater accuracy in further stratifying the prognosis of HCC patients belonging to Child–Pugh class A [14,52,53]. Two recent meta-analyses including HCC patients [16,51] reported a higher predictive value of the ALBI grade compared to the Child–Pugh class for stratifying patient survival. 

Indeed, higher ALBI grades were associated with poor overall survival (OS) (HR = 2.060, 95% CI: 1.909–2.211, *p* = 0.000) [16] even in HCC patients undergoing LR [51]. Another recent meta-analysis [54] confirmed that the ALBI grade was able to better stratify the prognosis of HCC patients undergoing treatments. Specifically, among Child–Pugh class A patients, those with ALBI grade 1 showed a higher OS rate compared to ALBI grade 2 [54], even after surgical resection. However, none of these previous pooled data analyses was focused on PHLF as the main outcome. 

Since liver function impairment is the main determinant of PHLF development and the vast majority of candidates to LR belonged to Child–Pugh class A [54,55], we expected that the ALBI grade could be a valuable tool for PHLF risk stratification. Our meta-analysis, evaluating a population almost entirely stratified as Child–Pugh class A (96.5%), confirmed its good performance in this setting, suggesting that a further stratification, over the Child–Pugh classification, could be safely and non-invasively applied in clinical practice without other time-consuming examinations. 

Further supporting the ALBI superiority in evaluating liver function and patient prognoses, one [36] of the studies included in the present meta-analysis showed that the ALBI score (AUC 0.745) was more accurate than the Child–Pugh classification (AUC 0.665), ICG R15 (AUC 0.668), and MELD score (AUC 0.649) in predicting PHLF. However, the MELD score was specifically designed for end-stage cirrhotic patients [56], and thus a low accuracy in predicting PHLF in compensated Child–Pugh A patients undergoing LR was expected. 

This meta-analysis has some weaknesses. The small number of studies included could have led to an underestimation of the association between ALBI and PHLF; however, we showed no publication bias and, using the ‘trim and fill’ methods to further strengthen our results, we found that no hypothetical studies were missing in our analysis. Concerning the reference standard, two studies [38,44] considered as case groups only PHLF grades B and C, thus, introducing a misclassification bias, in particular for the definition of patients with PHLF, which could have been underestimated. 

However, we carried out a sensitivity analysis after excluding these two studies [38,44], which showed no significant differences with our initial results. Most of the studies included considered the overall rate of PHLF, without distinguishing between PHLF grades; therefore, it was not possible to further stratify according to the presence of clinically significant PHLF, which could be more relevant in clinical practice [7]. At the same time, there were insufficient data to perform a subgroup analysis according to the extent of LR, which represents one of the other most relevant risk factors for PHLF. 

Another weakness of our meta-analysis was the substantial heterogeneity between the studies included. Among the differences found within the included studies, one study [44] included a small subgroup of patients undergoing LR for reasons other than HCC. We performed a sensitivity analysis excluding this latter study [44], showing no significant differences in the estimated OR. In addition, we found variability in the extension of hepatectomy, which, as mentioned above, could have also influenced the occurrence rate of PHLF. 

However, we further addressed the heterogeneity by performing a univariate meta regression analysis that showed that none of the variables tested, including an extension of the hepatectomy, was able to explain the heterogeneity found. Last, most studies of the included in the present meta-analysis were carried out in China, and thus reported on HBV patients. The race and the etiology of the underlying liver disease may influence the tumor biology, thus, adding a further bias to the surgical outcomes.

Our meta-analysis has several strengths supporting its value, as it provided for first-time pooled estimates of studies assessing the association between the ALBI grade and the occurrence of PHLF. Among the strengths of this meta-analysis, we performed a comprehensive literature search that minimized the risk of missing studies and, in the case of missing data, we contacted the authors to improve the data extraction. Another strength of our meta-analysis was the good methodological quality of the studies included. Despite the inclusion of only seven studies, we were able to include a large number of patients (5377) who underwent LR, with a reported PHLF rate of 13.4%. Of these, 69.9% were clinically significant, which is, thus, in line with other studies reporting the occurrence of PHLF [5,57]. 

In conclusion, our results provide additional evidence that the pre-operative ALBI grade is associated with the occurrence of PHLF. This has prognostic value for predicting this severe complication. The ALBI grade is a non-invasive, blood-test-based simple score that is able to further stratify the individual prognosis of chronic liver disease patients undergoing LR and reduce post-operative complications, such as PHLF. Further well-designed high-quality studies for evaluating the accuracy of the ALBI grade in the prediction of PHLF are needed.

## Figures and Tables

**Table 1:** The characteristics of the included studies in the systematic review and meta-analysis.

Author, Year	Country	Design of the Study	Total Pts	Age	Sex (Male), n. (%)	Etiology, %	Child Pugh, n. (%)	Extent of Hepatectomy n. (%)	Outcome	ALBI Grade n., (%)	Total PHLF
Wang, 2016 [43]	China	Retrospective	1242	>60: 223 (18%)	1072 (86.3)	HBV 85.3%	A 1189 (95.7), B 53 (4.3), C 0 (0)	Minor 975 (78.5), Major 267 (21.5)	PHLF A + B + C	ALBI 1, 850 (68.4), ALBI 2, 390 (31.4), ALBI 3, 2(0⋅2)	166, Grade A 58, Grade B 91, Grade C 17
Chong, 2018 [45]	China	Retrospective	396	59.7 ^c^	334 (84.3)	HBV 80.3%	A 397 (97.7), B 9 (2.3), C 0 (0)	Minor 243 (61.4), Major 153 (38.6)	PHLF A + B + C	ALBI 1, 302 (76.25), ALBI 2, 93 (23.5), ALBI 3, 1 (0.25)	109, Grade A 52, Grade B/C 57
Zhang, 2018 [40]	China	Retrospective	338	52 [44–66] ^b^	299 (88.5)	HBV 82.2%	A 308 (91.1), B 30 (8.9), C 0 (0)	N/A	PHLF A + B + C	ALBI 1, 134 (39.6), ALBI 2, 198 (58.6), ALBI 3, 6 (1.8)	26, Grade A 8, Grade B 13, Grade C 5
Zou, 2018 [36]	China	Retrospective	473	52 (18–77) ^a^	411 (86.9)	HBV 85.4%	A 427 (90.3), B 46 (9.7), C 0 (0)	Minor 356 (75,3), Major 117 (24.7)	PHLF A + B + C	ALBI 1, 189 (40), ALBI 2, 282 (59.6), ALBI 3, 2 (0.4)	50
Lu, 2019 [38]	China	Retrospective	2038	> 50: 948 (46.5%)	1810 (88.8)	HBV 88.9%	A 2038 (100), B 0 (0), C 0 (0)	Minor 1501 (73.7), Major 537 (26.3)	PHLF B + C	ALBI 1, 1570 (77), ALBI 2, 468 (23), ALBI 3, 0 (0)	196
Russolillo, 2019 [29]	Italy	Retrospective	400	70 (24–86) ^a^	339 (84.8)	Mixed (HCV 40%)	A 385 (96.25), B 15 (3.75), C 0 (0)	Minor 299 (74.7), Major 101 (25.3)	PHLF A + B + C	ALBI 1, 208 (52), ALBI 2, 188 (47), ALBI 3, 4 (1)	82, Grade A 48, Grade B/C 34
Sposito, 2020 [44]	Italy	Prospective	490	68.6 [61.4–74.7] ^b^	360 (73.5)	Mixed (HCV 59%)	A 463 (94.5), B 27 (5.5), C 0 (0)	Minor 457 (93.3), Major 33 (6.7)	PHLF B + C	ALBI 1, 217 (44.3), ALBI 2, 268 (54.7), ALBI 3, 5 (1.0)	89

Abbreviations: pts: patients; n.: number; ALBI: Albumin-Bilirubin score; HBV: Hepatitis B Virus; HCV: Hepatitis C Virus; N/A: Not Available; and PHLF: Post-Hepatectomy Liver Failure. ^a^ = median, (range); ^b^ = median, [interquartile range]; and ^c^ = median.

**Table 2:** The risk of bias and applicability concerns of the included studies.

Study	Patient Selection		Index Test		Reference Standard		Flow and Timing
	Risk of Bias	Concerns about Applicability	Risk of Bias	Concerns about Applicability	Risk of Bias	Concerns about Applicability	Risk of Bias
Wang, 2016 [43]	L	L	L	L	U	L	U
Chong, 2018 [45]	L	L	L	L	U	L	U
Zhang, 2018 [40]	U	L	L	L	U	L	U
Zou, 2018 [36]	U	L	L	L	U	L	U
Lu, 2019 [38]	U	L	L	L	U	H	U
Russolillo, 2019 [29]	L	L	L	L	U	L	U
Sposito, 2020 [44]	L	L	L	L	U	H	U

L, low; H, high; and U, unclear.

**Table 3:** Results of the univariable meta-regression analysis.

Covariates	Number of Studies	Beta Coefficient ± SE	Adjusted R^2^ (%)	*p* Value	*p* Value ± SE after Montecarlo Permutation
Country	7	0.308 ± 0.369	−2.03	0.371	0.432 ± 0.007
Age	5	0.871 ± 0.074	29.67	0.202	0.283 ± 0.006
Sex (Male)	7	1.097 ± 0.126	−9.15	0.460	0.485 ± 0.007
Child–Pugh A	7	1.014 ± 0.017	−6.57	0.442	0.437 ± 0.007
Major hepatectomy	5	0.956 ± 0.046	−3.46	0.424	0.316 ± 0.007
PHLF definition	7	1.462 ± 1.900	−21.97	0.782	0.852 ± 0.005

SE = Standard Error; R^2^ = Relative reduction in between-study variance: the value indicates the proportion of between study variance explained by covariate; and PHLF: Post-Hepatectomy Liver Failure.

## Data Availability

The data presented in this study are openly available in Medline and Embase.

## Supplementary Materials

The following are available online at https://www.mdpi.com/article/10.3390/jcm10092011/s1, Supplementary material 1: PRISMA Checklist; Supplementary material 2: Electronic search strategy of the literature, Supplementary material 3: Criteria for rating the methodological quality of the included studies (QUADAS-2), Supplementary material 4: Methodological quality assessment of the included studies.
