# Supplementary material for: Prognostic Value of the Albumin-Bilirubin Grade for the Prediction of Post-Hepatectomy Liver Failure: A Systematic Review and Meta-Analysis
Source: J Clin Med. 2021 May 8;10(9):2011. doi: 10.3390/jcm10092011 (PMC8125808; doi:10.3390/jcm10092011)
Supplement: Supplementary file 1 [file jcm-10-02011-s001.zip › jcm-1135284-supplementary.pdf]

1. Supplementary material 1: PRISMA Checklist

2. **Section/topic .**	3. **#.**	4. **Checklist item .**	5. **Reported on page # ..**
6. **TITLE ..**			7. .
8. Title .	9. 1.	10. Identify the report as a systematic review, meta-analysis, or both. .	11. 1..
12. **ABSTRACT ..**			13. .
14. Structured summary .	15. 2.	16. Provide a structured summary including, as applicable: Background; objectives; data sources; study eligibility criteria, participants, and interventions; study appraisal and synthesis methods; results; limitations; conclusions and implications of key findings; systematic review registration number. .	17. 2..
18. **INTRODUCTION ..**			19. .
20. Rationale .	21. 3.	22. Describe the rationale for the review in the context of what is already known. .	23. 3..
24. Objectives .	25. 4.	26. Provide an explicit statement of questions being addressed with reference to participants, interventions, comparisons, outcomes, and study design (PICOS). .	27. 3..
28. **METHODS ..**			29. .
30. Protocol and registration .	31. 5.	32. Indicate if a review protocol exists, if and where it can be accessed (e.g., Web address), and, if available, provide registration information including registration number. .	33. 4..
34. Eligibility criteria .	35. 6.	36. Specify study characteristics (e.g., PICOS, length of follow-up) and report characteristics (e.g., years considered, language, publication status) used as criteria for eligibility, giving rationale. .	37. 4-5..
38. Information sources .	39. 7.	40. Describe all information sources (e.g., databases with dates of coverage, contact with study authors to identify additional studies) in the search and date last searched. .	41. 4..
42. Search .	43. 8.	44. Present full electronic search strategy for at least one database, including any limits used, such that it could be repeated. .	45. 4..
46. Study selection .	47. 9.	48. State the process for selecting studies (i.e., screening, eligibility, included in systematic review, and, if applicable, included in the meta-analysis). .	49. 4-5..
50. Data collection process .	51. 10.	52. Describe method of data extraction from reports (e.g., piloted forms, independently, in duplicate) and any processes for obtaining and confirming data from investigators. .	53. 4-5..
54. Data items .	55. 11.	56. List and define all variables for which data were sought (e.g., PICOS, funding sources) and any assumptions and simplifications made. .	57. 4-5..
58. Risk of bias in individual studies .	59. 12.	60. Describe methods used for assessing risk of bias of individual studies (including specification of whether this was done at the study or outcome level), and how this information is to be used in any data synthesis. .	61. 5-6..

62. Summary measures .	63. 13.	64. State the principal summary measures (e.g., risk ratio, difference in means). .	65. 5-6..
66. Synthesis of results .	67. 14.	68. Describe the methods of handling data and combining results of studies, if done, including measures of consistency (e.g., I^2) for each meta-analysis. .	69. 5-6..

70. Page 1 of 2 .

71. **Section/topic .**	72. **#.**	73. **Checklist item .**	74. **Reported on page # ..**
75. Risk of bias across studies .	76. 15.	77. Specify any assessment of risk of bias that may affect the cumulative evidence (e.g., publication bias, selective reporting within studies). .	78. 6-7..
79. Additional analyses .	80. 16.	81. Describe methods of additional analyses (e.g., sensitivity or subgroup analyses, meta-regression), if done, indicating which were pre-specified. .	82. 6-7..
83. **RESULTS ..**			84. .
85. Study selection .	86. 17.	87. Give numbers of studies screened, assessed for eligibility, and included in the review, with reasons for exclusions at each stage, ideally with a flow diagram. .	88. 8..
89. Study characteristics .	90. 18.	91. For each study, present characteristics for which data were extracted (e.g., study size, PICOS, follow-up period) and provide the citations. .	92. 8..
93. Risk of bias within studies .	94. 19.	95. Present data on risk of bias of each study and, if available, any outcome level assessment (see item 12). .	96. 9..
97. Results of individual studies .	98. 20.	99. For all outcomes considered (benefits or harms), present, for each study: (a) simple summary data for each intervention group (b) effect estimates and confidence intervals, ideally with a forest plot. .	100. 8-9..
101. Synthesis of results .	102. 21.	103. Present results of each meta-analysis done, including confidence intervals and measures of consistency. .	104. 9-10..
105. Risk of bias across studies .	106. 22.	107. Present results of any assessment of risk of bias across studies (see Item 15). .	108. 9-10..
109. Additional analysis .	110. 23.	111. Give results of additional analyses, if done (e.g., sensitivity or subgroup analyses, meta-regression [see Item 16]). .	112. 9-10..
113. **DISCUSSION ..**			114. .
115. Summary of evidence .	116. 24.	117. Summarize the main findings including the strength of evidence for each main outcome; consider their relevance to key groups (e.g., healthcare providers, users, and policy makers). .	118. 10..
119. Limitations .	120. 25.	121. Discuss limitations at study and outcome level (e.g., risk of bias), and at review-level (e.g., incomplete retrieval of identified research, reporting bias). .	122. 12..
123. Conclusions .	124. 26.	125. Provide a general interpretation of the results in the context of other evidence, and implications for future research. .	126. 12..
127. **FUNDING ..**			128. .
129. Funding .	130. 27.	131. Describe sources of funding for the systematic review and other support (e.g., supply of data); role of funders for the systematic review. .	132. 1..

133. *From:* Moher D, Liberati A, Tetzlaff J, Altman DG, The PRISMA Group (2009). Preferred Reporting Items for Systematic Reviews and Meta-Analyses: The PRISMA Statement. PLoS Med 6(7): e1000097. doi:10.1371/journal.pmed1000097.
134. For more information, visit: **www.prisma-statement.or.**
135. Supplementary material 2. Electronic search strategy of the literature. .
136. MEDLINE via PubMed (n. 47).
137. ('ALBI' OR 'albumin bilirubin') AND ('PHLF' OR 'post operative liver failure' OR 'post hepatectomy liver failure') AND ('liver resection' OR 'hepatectomy' OR 'hepatic resection').
138. Ovid Embase (n. 33).
139. ('albi' OR 'albumin bilirubin') AND ('phlf' OR 'post operative liver failure' OR 'post hepatectomy liver failure'/exp OR 'post hepatectomy liver failure') AND ('liver resection'/exp OR 'liver resection' OR 'hepatectomy'/exp OR 'hepatectomy' OR 'hepatic resection'/exp OR 'hepatic resection').
140. Scopus (n. 130).
141. ('ALBI' OR 'albumin bilirubin') AND ('PHLF' OR 'post operative liver failure' OR 'post hepatectomy liver failure') AND ('liver resection' OR 'hepatectomy' OR 'hepatic resection').
142. Supplementary material 3. Criteria for rating the methodological quality of the included studies (QUADAS-2).
143. **Domain: Patient selection.**
144. **Risk of bias:.**
145. Question 1: Was a consecutive or random sample of patients enrolled? 'Yes' if consecutive.
146. or random sampling was explicitly stated, 'no' if non-consecutive or convenience sampling.
147. was performed, and 'unclear' if not reported.
148. Question 2: Was a case-control study avoided? 'Yes' if the study enrolled participants with no previously known risk of post-hepatectomy liver failure (cohort type study), "no" if the study enrolled participants with known risk of post-hepatectomy liver failure (case-control study), and 'unclear' if not reported.
149. Question 3: Did the study avoid inappropriate exclusions? .
150. "Yes" if all patients were included, without concerning their risk of developing post-hepatectomy liver failure, "no" if sub-groups of patients with increased or reduced risk of developing post-hepatectomy liver failure were excluded, "unclear", if data reported did not allow to make a judgment. .

.

151. **Concerns regarding applicability:.**
152. Are there concerns that the included patients and setting do not match the review question? 'No' if all participants had need for hepatectomy for hepatocellular carcinoma and received a pre-operative ALBI grade assessment, 'yes' if any participants did not have need for hepatectomy for hepatocellular carcinoma or pre-operative ALBI assessment has not been performed, 'unclear' if not clearly stated. Hepatocellular carcinoma must be surgery indication at least in >90% of patients included.
153. **Domain: Index test.**

.

154. **Risk of bias:.**
155. Question 1: Were the index test results interpreted without knowledge of the results of the reference standard? 'Yes' or 'no' if clearly stated, 'unclear' if it is not specified. .
156. **Concerns regarding applicability:.**
157. Are there concerns that the index test, its conduct, or its interpretation differ from the review question? 'No' if ALBI assessment and grading was conducted and interpreted according to its first definition by Johnson et al.[14], 'yes' if otherwise.
158. **Domain: Reference standard.**

.

159. **Risk of bias.**
160. Question 1: Is the reference standard likely to correctly classify the target condition? .
161. 'Yes' if the target condition was classified as the presence of PHLF (including grade A, B and/or C) according to International Study Group of Liver Surgery (ISGLS)[7], 'no' if otherwise.

.

162. Question 2: Were the results from the reference standard interpreted without knowledge of the results from the index test? .
163. 'Yes' or 'no' if clearly stated, 'unclear' if not reported.

.

164. **Concerns regarding applicability:.**
165. Are there concerns that the target condition as defined by the reference standard does not match the review question? **.**

166. **"**No" if the target condition was defined as diagnosis of PHLF, including grade A, B and/or C[7]; "yes" if the definition of the target condition was different (ex. if only clinically relevant PHLF of grade B/C), "unclear" if not clearly stated.

.

.

167. Domain: Flow and timing.

.

168. Question 1: Was there an appropriate interval between the index test and reference standard?.

169. 'Yes' if the ALBI assessment was performed within one month before the liver surgery, 'no' if otherwise, "unclear" if not clearly specified.

.

170. Question 2: Were all patients included in the analysis? .

171. 'Yes' if the number of participants enrolled does not differ from that of participants included in the results, 'no' if participants who were enrolled in the study were excluded from the analysis, and 'unclear' if data were not clear about it.

.

172. **Rules for producing an overall risk of bias rating for each domain:.**

- If all signalling questions within the domain are answered 'yes' then the risk of bias for this domain is rated 'low'.
- If at least one signalling question within the domain is answered 'no' then the risk of bias for this domain is rated 'high'.
- If at least one signalling question within the domain is answered 'unclear' while the remaining signalling questions are answered 'yes' then the risk of bias is rated 'unclear'.

173. Supplementary material 4. Methodological quality assessment of the included studies.

174. ..	175.<br>176. Risk of Bias.								177. Concerns regarding applicability..		
178. Study.	179. Patient selection.			180. Index test.	181. Reference standard.		182. Flow and timing.		183. Patient selection.	184. Index test.	185. Referenc e standard..
186.	187. Q1	188. Q2	189.<br>190. Q3	191. Q1	192. Q1	193. Q2	194. Q1	195. Q2	196.	197.	198.
199. Wang, 2016[43]	200. Yes	201. Yes	202. Yes	203. Yes	204. Yes	205. Unclear	206. Unclear	207. Yes	208. No	209. No	210. No
211. Chong, 2018[45]	212. Yes	213. Yes	214. Yes	215. Yes	216. Yes	217. Unclear	218. Unclear	219. Yes	220. No	221. No	222. No
223. Zhang, 2018[40]	224. Unclear	225. Yes	226. Yes	227. Yes	228. Yes	229. Unclear	230. Unclear	231. Yes	232. No	233. No	234. No
235. Zou, 2018[36]	236. Unclear	237. Yes	238. Yes	239. Yes	240. Yes	241. Unclear	242. Unclear	243. Yes	244. No	245. No	246. No
247. Russolillo, 2019[29]	248. Yes	249. Yes	250. Yes	251. Yes	252. Yes	253. Unclear	254. Unclear	255. Yes	256. No	257. No	258. No
259. Lu, 2019[38]	260. Unclear	261. Yes	262. Yes	263. Yes	264. Yes	265. Unclear	266. Unclear	267. Yes	268. No	269. No	270. Yes
271. Sposito, 2020[44]	272. Yes	273. Yes	274. Yes	275. Yes	276. Yes	277. Unclear	278. Unclear	279. Yes	280. No	281. No	282. Yes

283. Abbreviations: Q: Question.
